# Deep learning radiomics models based on contrast-enhanced transrectal ultrasound for predicting distant metastasis in rectal cancer

**DOI:** 10.3389/fonc.2026.1671887

**Published:** 2026-02-09

**Authors:** Zhiyuan Xia, Lidan Liu, Haining Chen, Yanling Mo, Yefu Shen, Xihua Xie, Ming Qiu, Cun Liao, Huanyu Cui, Sen Zhang

**Affiliations:** 1 Department of Colorectal & Anal Surgery, The First Affiliated Hospital of Guangxi Medical University, Nanning, China; 2 Guangxi Key Laboratory of Enhanced Recovery After Surgery for Gastrointestinal Cancer, Nanning, China; 3 Guangxi Reproductive Medical Center, The First Affiliated Hospital of Guangxi Medical University, Nanning, China; 4 Department of Ultrasound, The First Affiliated Hospital of Guangxi Medical University, Nanning, China

**Keywords:** contrast-enhanced transrectal ultrasound, deep learning, distant metastasis, radiomics, rectal cancer

## Abstract

**Objective:**

Rectal cancer is a common malignant tumor, and the presence of distant metastasis is critically important for determining treatment strategies. This study aimed to develop a deep learning radiomics model based on contrast-enhanced transrectal ultrasound (CETRUS) imaging to predict distant metastasis in patients with rectal cancer.

**Methods:**

We retrospectively analyzed the clinical data and CETRUS imaging of 878 patients with rectal cancer treated at The First Affiliated Hospital of Guangxi Medical University. Univariate and multivariate logistic regression analyses were performed to identify relevant clinical variables. Deep learning radiomics features were extracted using a pretrained DenseNet201 model and subsequently selected via the Mann–Whitney U test, Spearman correlation analysis, and least absolute shrinkage and selection operator regression. Separate models were constructed based on clinical data, two-dimensional ultrasound (TDUS), color Doppler ultrasound (CDUS), and contrast-enhanced ultrasound (CEUS) imaging. The optimal deep learning radiomics model was then combined with the clinical model to develop an integrated predictive model.

**Results:**

The clinical prediction model achieved area under the curve (AUC) values of 0.631 and 0.604 in the training and test cohorts, respectively. Among the three deep learning radiomics models, the CEUS model demonstrated the best performance, with AUC of 0.950 and 0.740 in the training and test cohorts, respectively. The TDUS model achieved AUC of 0.935 and 0.586, while the CDUS model yielded AUC of 0.805 and 0.521. The integrated model combining the clinical and contrast-enhanced ultrasound radiomics models achieved AUC of 0.947 and 0.749 in the training and test cohorts, respectively.

**Conclusion:**

The clinical-deep learning radiomics model based on CETRUS showed promising predictive performance in assessing distant metastasis in rectal cancer patients. This approach has the potential to assist clinicians in developing personalized patient management strategies, pending further validation to confirm its clinical applicability.

## 1 Introduction

Rectal cancer is a common malignancy of the gastrointestinal tract. Compared with gastric cancer, which has a 5-year survival rate of 35.7%, and pancreatic cancer, with a 5-year survival rate of 13%, rectal cancer demonstrates a relatively favorable prognosis, with a 5-year survival rate of approximately 67% (1–6). However, clinical data indicate that nearly 20% of patients with colorectal cancer present with distant metastasis at initial diagnosis. These patients generally have a poor prognosis, with a 5-year survival rate of only 16% (2, 7). Notably, there are fundamental differences in therapeutic strategies between early-stage and advanced rectal cancer (8). Therefore, accurately identifying the presence of distant metastases prior to treatment planning is essential for devising precise and effective treatment strategies.

Contrast-enhanced transrectal ultrasound (CETRUS) is an effective imaging modality for the evaluation of rectal diseases, particularly for local staging of rectal cancer. The diagnostic accuracy of CETRUS for T staging of rectal cancer has been reported to reach 71.88%, which is significantly higher than that of MRI (51.56%) (9–12). However, CETRUS is unable to evaluate the presence of distant metastasis. Radiomics enables the extraction and analysis of quantitative imaging features that are imperceptible to the human eye and are often potentially associated with the biological behavior of tumors (13). To date, radiomics studies based on imaging modalities such as computed tomography (CT) and magnetic resonance imaging (MRI) have demonstrated promising predictive performance in evaluating distant metastases in rectal cancer (14, 15). However, no studies have yet been reported on the use of radiomics derived from CETRUS for predicting distant metastases in this context.

With the continuous development of radiomics techniques, an increasing variety of methods have been employed to extract radiomic features. Among these approaches, deep learning has emerged as a powerful tool for feature extraction due to its high degree of automation, strong feature representation capabilities, excellent adaptability to high-dimensional and complex data, superior generalization and transferability, and its capacity for seamless integration with clinical tasks (13, 16).

Therefore, in this study, we aimed to predict distant metastasis in rectal cancer patients by developing a clinical prediction model based on patient clinical data, as well as deep learning radiomics models derived from CETRUS, including two-dimensional ultrasound (TDUS), color Doppler ultrasound (CDUS), and contrast-enhanced ultrasound (CEUS). The optimal deep learning radiomics model was subsequently combined with the clinical predictive model to create a combined model. Additionally, a nomogram was constructed to facilitate visualization of the predictive results.

## 2 Materials and methods

### 2.1 Population

This study was granted ethical approval by the institutional review board of The First Affiliated Hospital of Guangxi Medical University, which was performed in accordance with the ethical standards of the 1964 Declaration of Helsinki. Informed consent was waived due to the retrospective nature of this study.

A retrospective analysis was conducted of the clinical data and CETRUS imaging data of patients with malignant rectal tumors treated at The First Affiliated Hospital of Guangxi Medical University between January 2021 and December 2023.

The inclusion criteria were as follows: 1. histopathological confirmation of malignant rectal tumors, with no prior antitumor treatment before imaging examinations; 2. performance of CETRUS after admission, with images stored in a standardized format; 3. completion of comprehensive imaging examinations after admission, including contrast-enhanced computed tomography (CT) of the chest, abdomen, and pelvis, as well as contrast-enhanced ultrasonography of the liver, to assess the presence of distant metastases; 4. no previous history of other malignancies.

The exclusion criteria were as follows: 1. poor-quality ultrasonographic images that compromised image segmentation and feature extraction; 2. multiple primary colorectal cancers; 3. incomplete clinical data.

**Figure 1:**
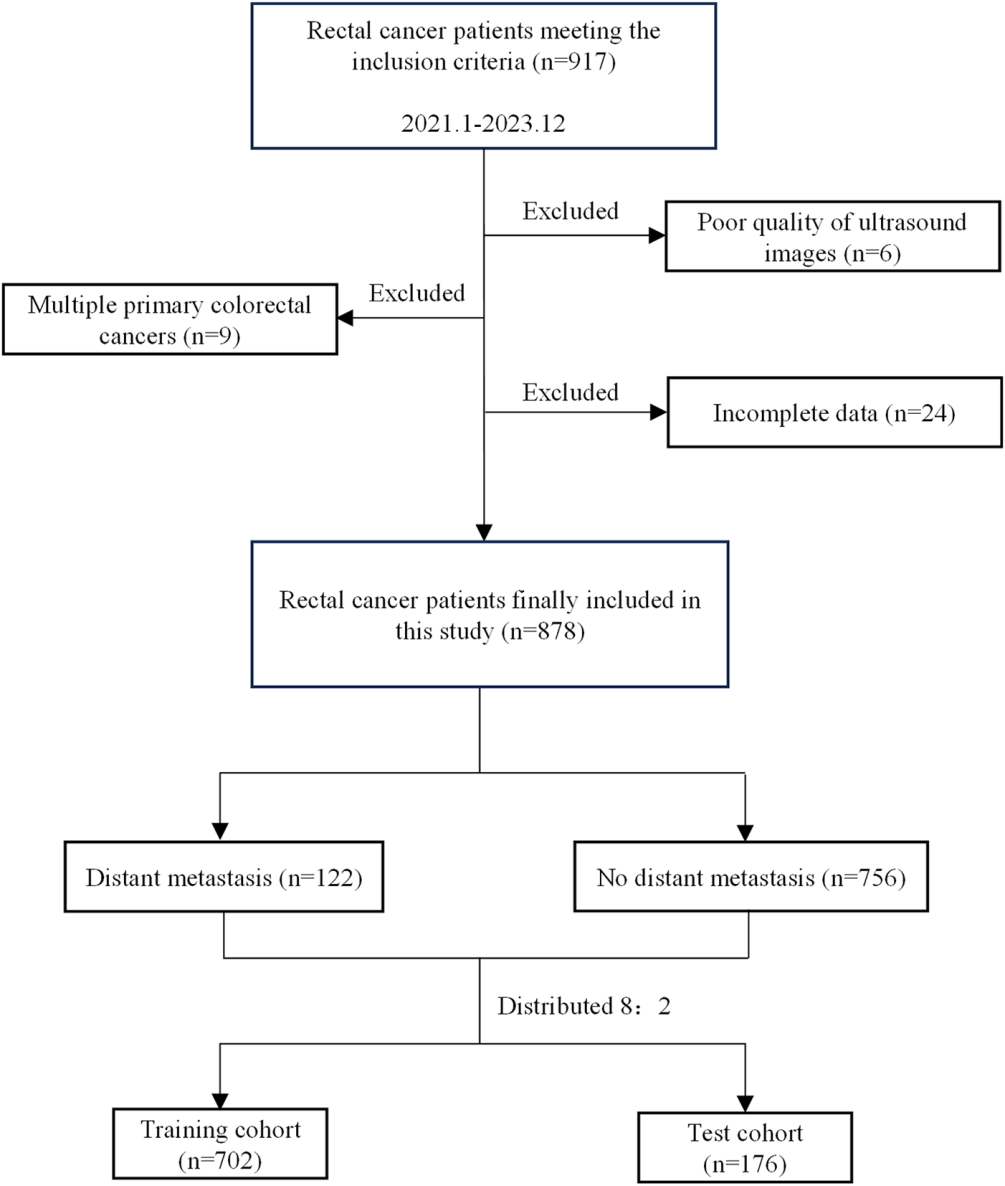
Flowchart illustrating patient enrollment, inclusion and exclusion criteria, and cohort allocation in this study.

A total of 878 patients with rectal cancer were ultimately included in this study. The dataset was randomly split 10 times at an 8:2 ratio to generate 10 independent training–test subset combinations, from which one split was randomly selected as the final training and test sets. The patient selection flowchart is shown in **Figure 1**.

### 2.2 Clinical outcomes

The primary outcome observed in this study was the occurrence of distant metastases. Two radiologists independently reviewed the contrast-enhanced CT scans of the chest, abdomen, and pelvis to determine the presence of distant metastases. In cases of disagreement between the two radiologists, a third radiologist reviewed the images to make the final determination.

Similarly, two sonographers independently assessed the contrast-enhanced ultrasonography of the liver to evaluate the presence of hepatic metastases. If discrepancies arose between the two sonographers, a third sonographer performed an additional review to reach a definitive conclusion.

When conflicting assessments were obtained between the contrast-enhanced CT and the liver contrast-enhanced ultrasonography, a multidisciplinary team discussion was conducted to establish the final diagnosis of distant metastases.

### 2.3 Ultrasonographic examination and image acquisition

All examinations were performed using a Canon Aplio 500 ultrasound diagnostic system equipped with an end-fire transrectal probe operating at a frequency range of 5–10 MHz. During the examination, patients were positioned in the left lateral decubitus position with hips and knees flexed. After insertion of the probe into the rectum, gray-scale mode was utilized to evaluate the tumor’s location, size, and depth of invasion, and TDUS images were acquired. Subsequently, the vascularity of the lesion was assessed, and CDUS images were collected. Finally, the system was switched to CEUS mode. SonoVue contrast agent (Bracco SpA, Italy) was administered intravenously via the patient’s forearm, and CEUS images were acquired 30 seconds after injection.

### 2.4 Image segmentation

**Figure 2:**
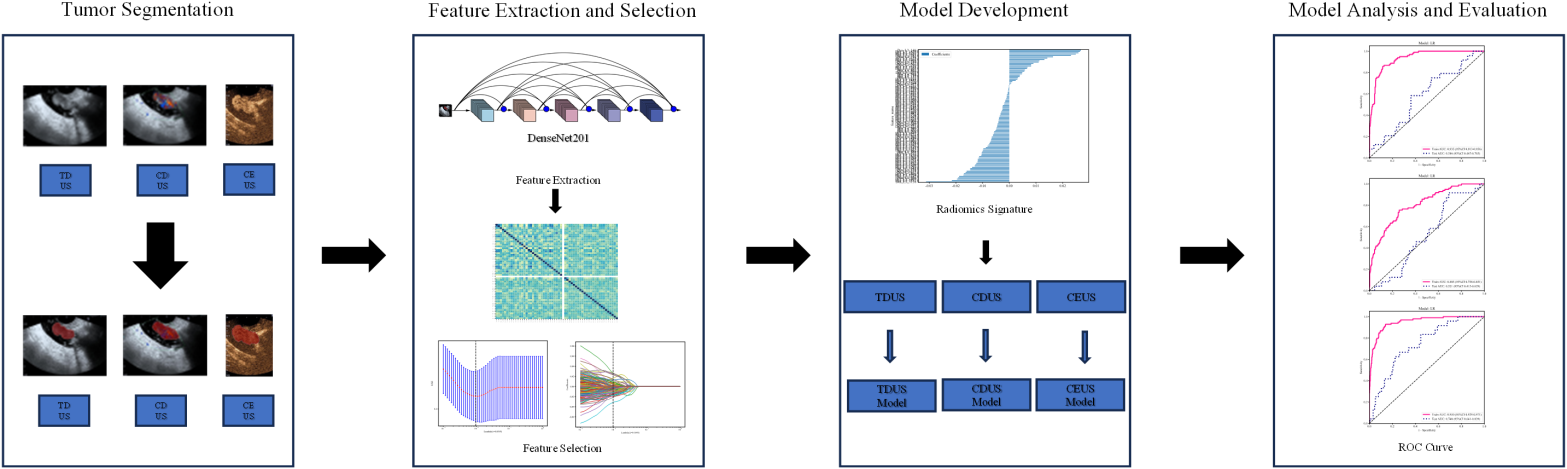
Workflow of the deep learning radiomics analysis, including image acquisition, tumor segmentation, feature extraction using DenseNet201, feature selection, model construction, and performance evaluation.

**Figure 3:**
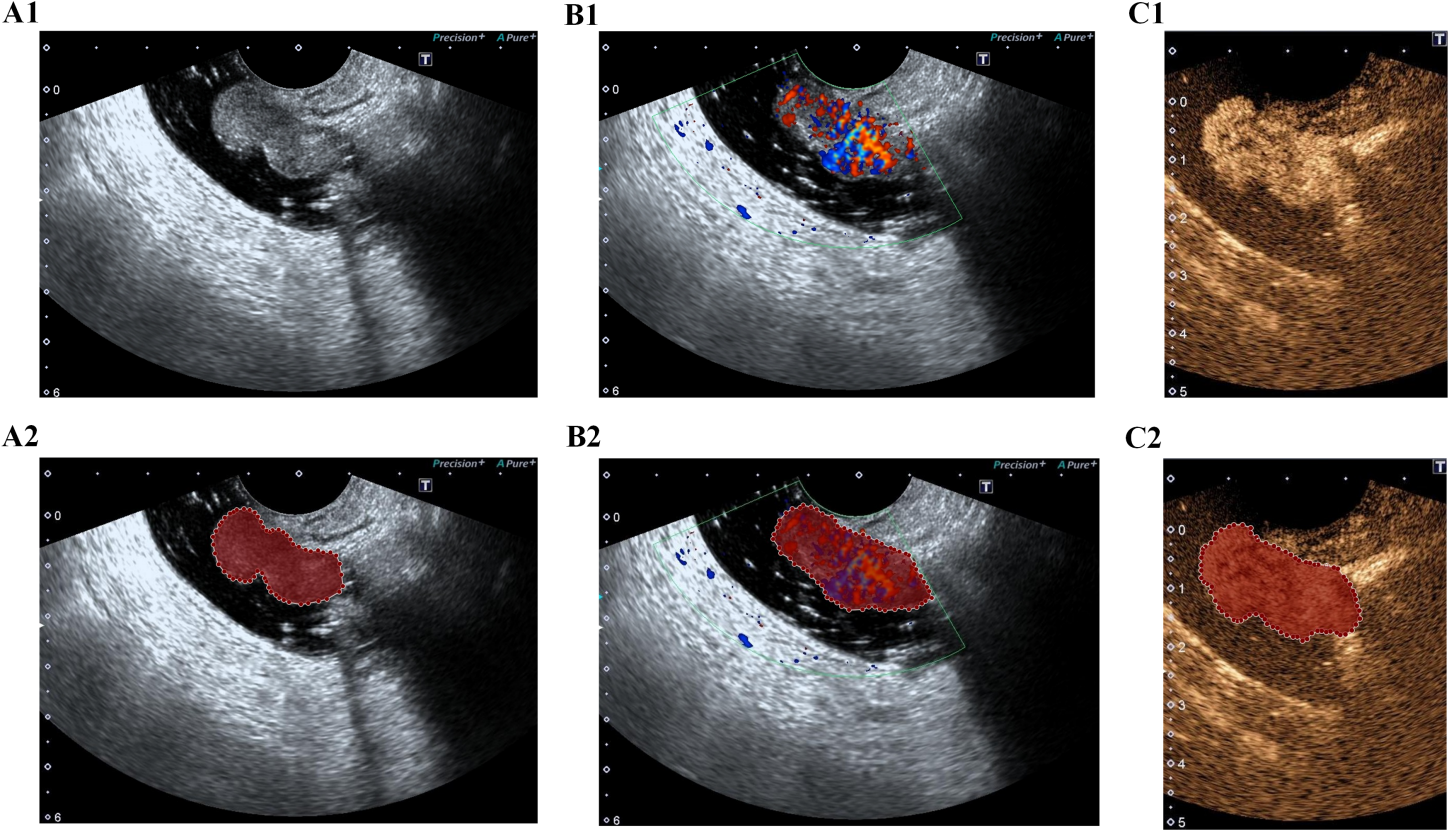
Representative examples of tumor segmentation on ultrasound images. **(A)** TDUS images before and after tumor segmentation. **(B)** CDUS images before and after segmentation. **(C)** CEUS images before and after segmentation.

The workflow of the radiomics study is illustrated in **Figure 2**. Images depicting the largest cross-sectional area of the tumor were imported into ITK-SNAP software (version 3.8.0). One sonographer, blinded to the clinical data, manually delineated the regions of interest (ROIs) along the tumor margins on the TDUS, CDUS, and CEUS images, respectively (**Figure 3**). Another sonographer performed ROI segmentation using the same method.

After both sonographers completed the ROI delineation, the consistency of the segmentations was evaluated by calculating the intraclass correlation coefficient (ICC). An ICC greater than 0.9 was considered indicative of good agreement. For cases with ICC values below 0.9, the final ROI was determined through consensus discussion involving the two ultrasound physicians and a third senior ultrasound specialist.

### 2.5 Deep learning radiomics feature extraction

In this study, a DenseNet201 model pretrained on the ImageNet dataset was employed to extract deep learning radiomics features. The pretrained DenseNet201 model comprises 201 layers and is capable of extracting multi-level and rich features. DenseNet201 is a deep convolutional neural network (CNN) characterized by a dense connectivity mechanism, whereby each layer receives inputs not only from the immediately preceding layer but also from all earlier layers. This architecture alleviates the vanishing gradient problem, enhances feature propagation, and substantially reduces the number of model parameters.

Features were extracted from the penultimate layer of DenseNet201 (features.adaptive_avg_pool2d). This layer is an adaptive average pooling layer that compresses the input feature maps into a fixed size (1×1), thereby transforming inputs of varying dimensions into uniformly sized outputs. Subsequently, the DenseNet201 model was applied separately to the TDUS, CDUS, and CEUS images to extract the corresponding deep learning radiomics features.

### 2.6 Selection of clinical variables and deep learning radiomics features

Univariate logistic regression analysis was performed on the clinical variables, and those with 95% confidence intervals (CIs) not including 1 were selected for further multivariate logistic regression analysis. In the multivariate analysis, variables with 95% CIs not including 1 were retained for subsequent model development.

For deep learning radiomics features, the Mann–Whitney U test was first applied to identify features significantly associated with distant metastasis (P < 0.05). Next, Spearman correlation analysis was conducted to remove redundant features; for any pair of features with a correlation coefficient greater than 0.9, only one was retained. Finally, least absolute shrinkage and selection operator (LASSO) regression was employed to select features with non-zero coefficients. The optimal deep learning radiomics features identified through this process were used for calculating the radiomics score. The radiomics score were used for model construction.

### 2.7 Model construction and performance evaluation

The selected clinical features and deep learning radiomics features were used separately to build logistic regression models, resulting in a clinical model, a TDUS model, a CDUS model, and a CEUS model.

The area under the receiver operating characteristic curve (AUC) was employed as the primary metric to evaluate the discriminative performance of each model. Additionally, accuracy, sensitivity, specificity, negative predictive value (NPV), and positive predictive value (PPV) were calculated to provide a comprehensive assessment of model effectiveness.

The deep learning radiomics model with the highest AUC in the test cohort was integrated with the clinical predictive model to construct a combined model. A nomogram was then developed to visualize the performance of the integrated model.

### 2.8 Statistical analysis

Statistical analyses were conducted using Python version 3.8.7. For continuous variables with normal distribution and homogeneity of variance, the t-test was applied, with data expressed as mean ± standard deviation (mean ± SD). For continuous variables with non-normal distribution or unequal variances, the Mann–Whitney U test was used, and data were presented as median and interquartile range (M [P25, P75]). Categorical variables were analyzed using the chi-square test and described as counts and percentages. A two-sided P value less than 0.05 was considered statistically significant.

## 3 Results

### 3.1 Comparison of clinical baseline characteristics

As presented in **Table 1**, among the 878 patients with rectal cancer, significant differences were observed between the distant metastasis group and the non-distant metastasis group in platelet count and C-reactive protein levels (P < 0.05). No statistically significant differences were found between the two groups in terms of sex, age, height, weight, smoking history, alcohol consumption, white blood cell count, red blood cell count, hemoglobin level, neutrophil percentage, lymphocyte percentage, monocyte percentage, or eosinophil percentage (P > 0.05).

**Table 1:** Univariate analysis of baseline clinical characteristics of rectal cancer patients in the training and test cohorts.

Variable	Distant metastasis group	Non-distant metastasis group	P
Sex			0.148
Male	84 (9.57%)	469 (53.41%)	
Female	38 (4.33%)	287 (32.69%)	
Age	60.00 (52.00, 68.00)	59.00 (53.00, 68.00)	0.845
Height	162.00 (156.00, 168.00)	162.00 (153.00, 167.00)	0.530
Weight	59.00 (52.50, 66.45)	59.90 (52.00, 66.70)	0.282
Smoking			0.756
Yes	41 (4.67%)	265 (30.18%)	
No	81 (9.23%)	491 (55.92%)	
Drinking			0.101
Yes	46 (5.24%)	229 (26.08%)	
No	76 (8.66%)	527 (60.02%)	
WBC	6.78 (5.90, 8.05)	6.78 (5.69, 8.13)	0.627
RBC	4.38 (3.99, 4.82)	4.36 (4.00, 4.71)	0.464
HGB	123.00 (110.85, 135.00)	122.55 (111.00, 134.05)	0.929
PLT	293.00 (239.00, 357.50)	267.10 (221.97, 321.70)	<0.001
NEU%	0.61 (0.56, 0.68)	0.61 (0.54, 0.68)	0.462
LYM%	0.26 (0.21, 0.31)	0.27 (0.21, 0.33)	0.187
MONO%	0.08 (0.07, 0.09)	0.08 (0.06, 0.09)	0.391
EO%	0.03 (0.02, 0.04)	0.02 (0.01, 0.04)	0.080
CRP	3.20 (1.42, 8.15)	2.10 (0.90, 5.40)	0.027

WBC, white blood cell count; RBC, red blood cell count; HGB, hemoglobin; PLT, platelet count; NEU, neutrophil; LYM, lymphocyte; MONO, monocyte; EO, eosinophil; CRP, C-reaction protein.

### 3.2 Selection of clinical variables

Univariate logistic regression analysis identified alcohol consumption (Odds Ratio (OR): 1.56, 95% Confidence Interval (CI): 1.00–2.42) and platelet count (OR: 1.00, 95% CI: 1.00–1.01) as variables associated with distant metastasis. Multivariate logistic regression analysis including these factors revealed that alcohol consumption (OR: 1.55, 95% CI: 1.00–2.43) and platelet count (OR: 1.00, 95% CI: 1.00–1.01) were independent predictors of distant metastasis, as detailed in **Table 2**.

**Table 2:** Univariate and multivariate logistic regression analyses of clinical variables associated with distant metastasis in rectal cancer patients.

Variable	Univariate analysis		Multivariate analysis	
	OR (95%CI)	P value	OR (95%CI)	P value
Sex	0.68 (0.42, 1.08)	0.10		
Age	1 (0.98, 1.02)	0.78		
Height	0.99 (0.97, 1.02)	0.59		
Weight	1 (0.98, 1.02)	0.64		
Smoking	0.95 (0.60, 1.48)	0.81		
Drinking	1.56 (1.00, 2.42)	0.05	1.55 (1.00, 2.43)	0.05
WBC	1.05 (0.96, 1.15)	0.29		
RBC	1.26 (0.90, 1.76)	0.18		
HGB	1 (0.99, 1.01)	0.95		
PLT	1 (1.00, 1.01)	<0.01	1.00 (1.00, 1.01)	<0.01
NEU%	0.94 (0.12, 7.41)	0.96		
LYM%	0.38 (0.04, 3.59)	0.40		
MONO%	29.43 (0.01, 103588.26)	0.42		
EO%	303.42 (0.42, 221223.91)	0.09		
CRP	1 (0.99, 1.01)	0.64		

WBC, white blood cell count; RBC, red blood cell count; HGB, hemoglobin; PLT, platelet count; NEU, neutrophil; LYM, lymphocyte; MONO, monocyte; EO, eosinophil; CRP, C-reaction protein.

### 3.3 Selection of deep learning radiomics features

**Figure 4:**
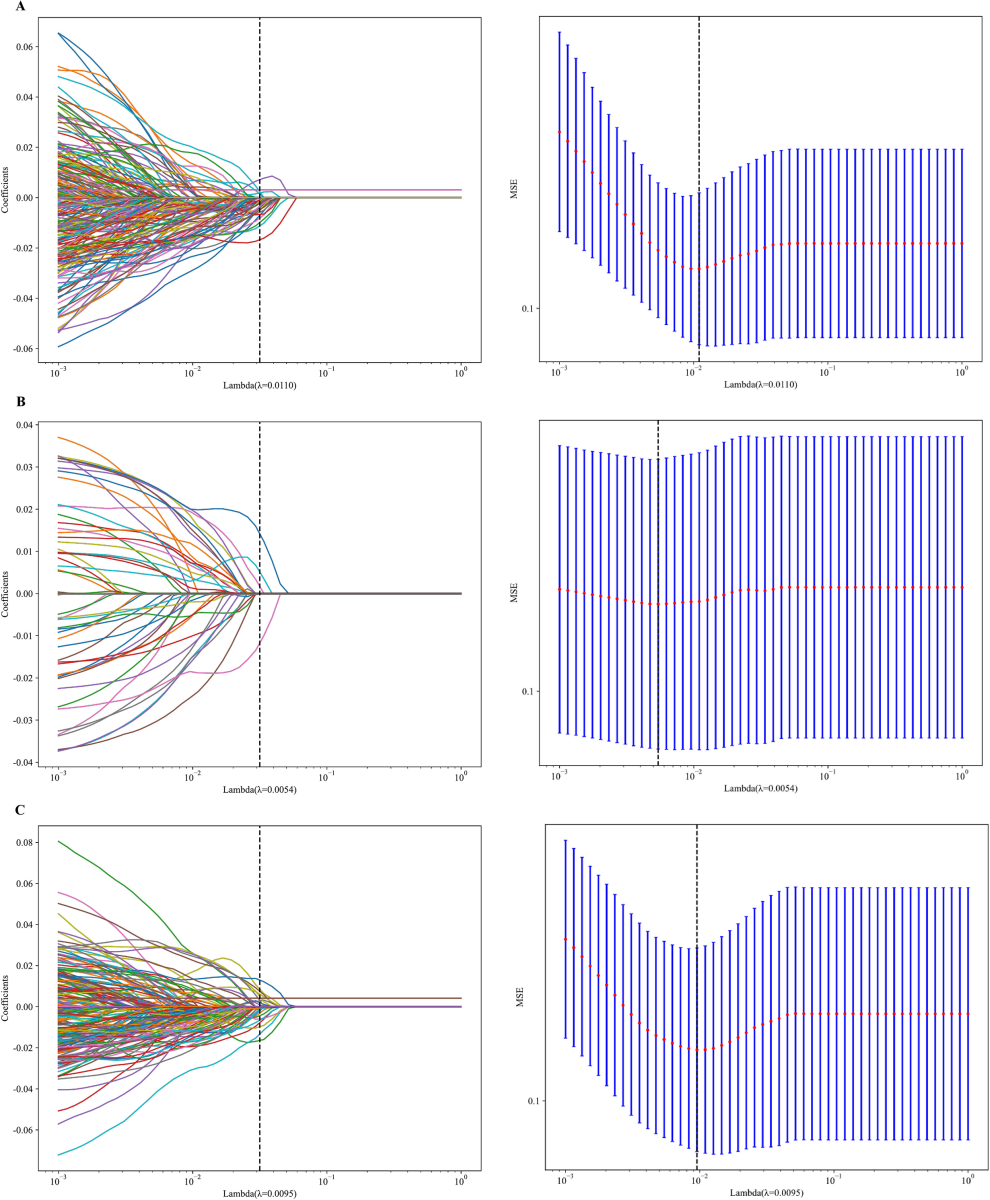
Feature selection using LASSO regression for deep learning radiomics features extracted by the DenseNet201 model. **(A)** TDUS-based features. **(B)** CDUS-based features. **(C)** CEUS-based features.

The mean ICC values for ROIs delineated by the two ultrasound physicians were 0.92 for TDUS, 0.91 for CDUS, and 0.94 for CEUS. A pretrained DenseNet201 model was applied to extract 1,920 deep learning radiomics features from the ROI on TDUS, CDUS, and CEUS images, respectively. After sequential feature selection using the Mann–Whitney U test, Spearman correlation analysis, and LASSO regression (**Figure 4**), 76 features from the TDUS, 42 features from the CDUS, and 101 features from the CEUS were retained for further analysis (**Supplementary Figure 1**).

### 3.4 Model construction and predictive performance evaluation

A logistic regression (LR) model was constructed using the selected clinical variables. The model achieved AUC values of 0.631 and 0.604 in the training and test cohorts, respectively. In the training cohort, the accuracy, sensitivity, specificity, positive predictive value (PPV), and negative predictive value (NPV) were 0.660, 0.571, 0.674, 0.221, and 0.906, respectively. In the test cohort, the corresponding values were 0.597, 0.708, 0.579, 0.210, and 0.926.

Separate LR models were developed based on the selected deep learning radiomics features from TDUS, CDUS, and CEUS images. The predictive performances of these models are summarized in **Table 3**. For the TDUS model, the AUC, accuracy, sensitivity, specificity, PPV, and NPV were 0.935, 0.880, 0.857, 0.884, 0.545, and 0.974 in the training cohort, and 0.586, 0.631, 0.583, 0.638, 0.203, and 0.907 in the test cohort, respectively.

**Table 3:** Predictive performance of the deep learning radiomics models based on TDUS, CDUS, and CEUS in the training and test cohorts.

Cohort	Model	AUC (95%CI)	Accuracy	Sensitivity	Specificity	PPV	NPV
Training cohort	TDUS	0.935 (0.913 - 0.956)	0.880	0.857	0.884	0.545	0.974
	CDUS	0.805 (0.758 - 0.852)	0.742	0.755	0.740	0.320	0.949
	CEUS	0.950 (0.929 - 0.971)	0.869	0.929	0.859	0.517	0.987
Test cohort	TDUS	0.586 (0.467 - 0.705)	0.631	0.583	0.638	0.203	0.907
	CDUS	0.521 (0.412 - 0.629)	0.392	0.917	0.309	0.173	0.959
	CEUS	0.740 (0.641 - 0.840)	0.727	0.667	0.737	0.286	0.933

For the CDUS model, the corresponding metrics in the training cohort were 0.805, 0.742, 0.755, 0.740, 0.320, and 0.949, while those in the test cohort were 0.521, 0.392, 0.917, 0.309, 0.173, and 0.959.

The CEUS model demonstrated superior performance, with AUC, accuracy, sensitivity, specificity, PPV, and NPV values of 0.950, 0.869, 0.929, 0.859, 0.517, and 0.987 in the training cohort, and 0.740, 0.727, 0.667, 0.737, 0.286, and 0.933 in the test cohort, respectively.

**Figure 5:**
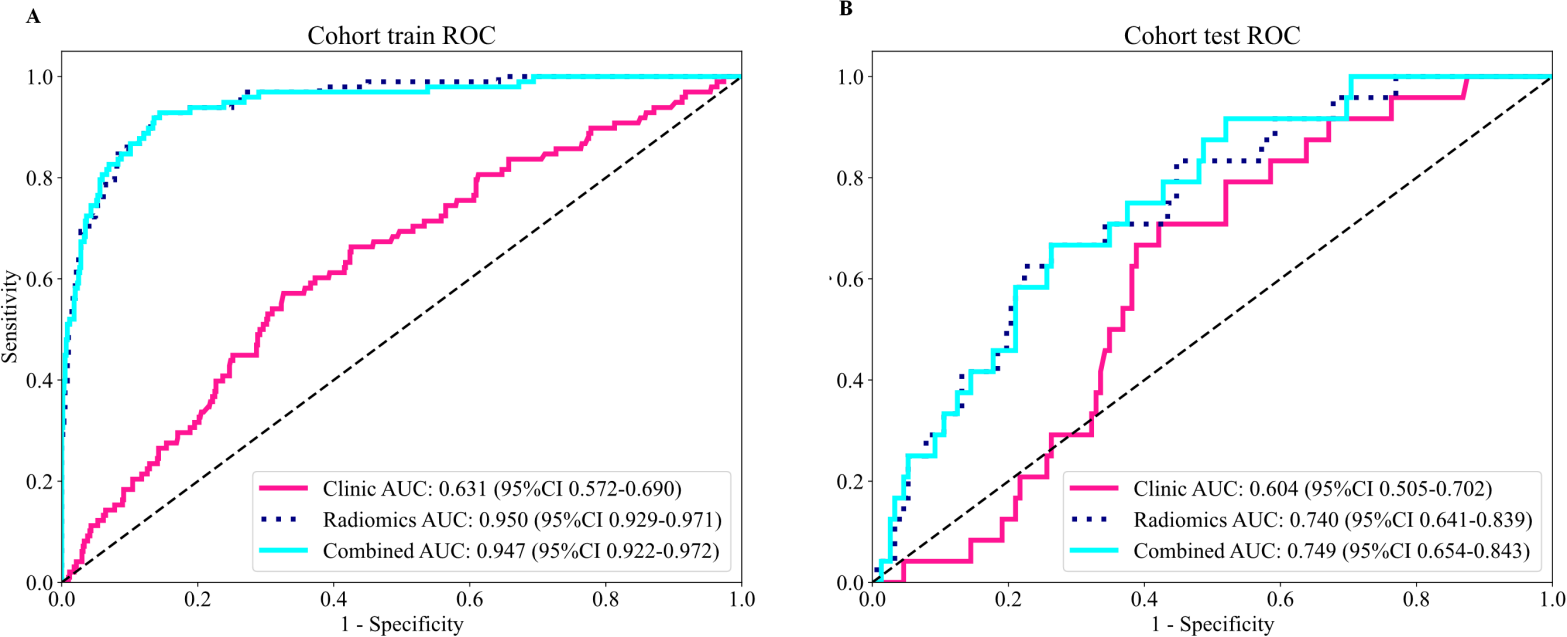
ROC curves and AUCs of the clinical model, CEUS-based deep learning radiomics model, and the integrated clinical–radiomics model in the test cohort.

Among the three deep learning radiomics models, the CEUS model exhibited the highest AUC. This model was therefore integrated with the clinical model to construct a combined predictive model. The integrated model yielded AUC, accuracy, sensitivity, specificity, PPV, and NPV values of 0.947, 0.866, 0.929, 0.856, 0.511, and 0.987 in the training cohort, and 0.749, 0.727, 0.667, 0.737, 0.286, and 0.933 in the test cohort, respectively (**Figure 5**; **Table 4**).

**Table 4:** Predictive performance of the clinical model, CEUS model and integrated model in the training and test cohorts.

Cohort	Model	AUC (95%CI)	Accuracy	Sensitivity	Specificity	PPV	NPV
Training cohort	Clinical model	0.631 (0.572-0.690)	0.660	0.571	0.674	0.221	0.906
	CEUS model	0.950 (0.929-0.971)	0.869	0.929	0.859	0.517	0.987
	Integrated model	0.947 (0.922-0.972)	0.866	0.929	0.856	0.511	0.987
Test cohort	Clinical model	0.604 (0.505-0.702)	0.597	0.708	0.579	0.210	0.926
	CEUS model	0.740 (0.641-0.840)	0.727	0.667	0.737	0.286	0.933
	Integrated model	0.749 (0.654-0.843)	0.727	0.667	0.737	0.286	0.933

### 3.5 Nomogram of the integrated model

**Figure 6:**
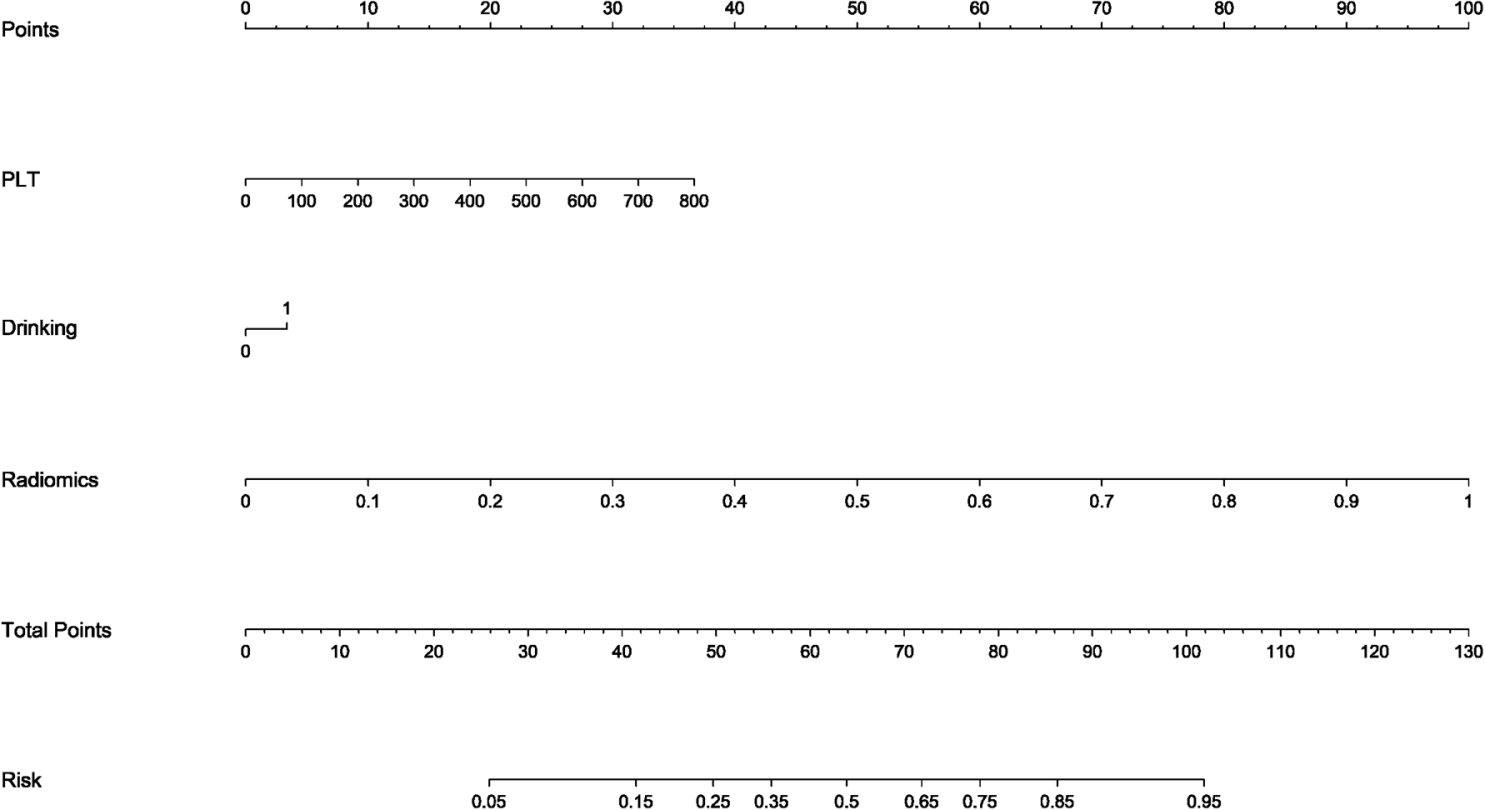
Nomogram constructed based on the integrated clinical–deep learning radiomics model for individualized prediction of distant metastasis in rectal cancer patients.

As shown in **Figure 6**, to enhance the clinical interpretability of the study findings, a nomogram was developed based on the final combined model. This visual tool enables clinicians to rapidly estimate an individual patient’s probability of distant metastasis by inputting their platelet count, alcohol consumption history, and CEUS radiomics score. The nomogram translates complex model outputs into accessible probability estimates, supporting informed clinical decision-making regarding the intensity of staging workup and treatment planning.

## 4 Discussion

Accurately determining the presence of distant metastasis in rectal cancer patients prior to treatment is crucial for devising appropriate therapeutic strategies. This approach not only prevents undertreatment in patients with metastasis but also avoids overtreatment in those without. In this study, we developed and validated a multiparametric model based on clinical data and deep learning radiomics to predict distant metastasis in rectal cancer patients. Our findings indicate that alcohol consumption and platelet count are independent risk factors for distant metastasis in rectal cancer. However, the clinical prediction model constructed solely on these factors demonstrated only moderate predictive performance. Among the deep learning radiomics models based on CETRUS imaging, the model derived from CEUS features exhibited the best performance. The integrated model combining the clinical prediction model with the CEUS deep learning radiomics model showed superior predictive accuracy for distant metastasis in rectal cancer.

Platelets, which are produced by bone marrow precursor cells and megakaryocytes, play a vital role in maintaining hemostasis and vascular integrity (17). Current studies have shown that elevated platelet counts are associated with tumor progression and poor prognosis (18–21). In addition, our study found that pre-treatment elevated platelet counts in rectal cancer patients serve as a risk factor for distant metastasis. McCarty et al. (22) further explored the mechanisms linking thrombocytosis to tumor metastasis, suggesting that platelets may promote distant metastasis by facilitating tumor thrombus formation and mediating interactions between tumor cells and platelet ligands.

Alcohol is one of the major risk factors for cancer development in humans and is strongly associated with adverse outcomes in various malignancies (23, 24). A meta-analysis has also revealed a correlation between consuming more than one alcoholic drink per day and an increased risk of colorectal cancer (25). Beyond its association with tumor occurrence and prognosis, this study found that a history of long-term alcohol consumption is related to distant metastasis in colorectal cancer patients. Further research is warranted to elucidate the underlying mechanisms involved.

The clinical model developed in this study demonstrated moderate predictive performance for distant metastasis in colorectal cancer patients, with AUC values of 0.631 in the training cohort and 0.604 in the test cohort. This limited performance may be attributed to the inclusion of only routine blood test and CRP indicators, and the relatively low odds ratios of the selected variables. When these limited clinical variables were combined with the hierarchically selected deep learning radiomics features to construct the integrated model, the incremental value of ultrasound-based deep learning radiomics may have been partially overestimated. Future studies should incorporate a broader range of clinically relevant variables associated with distant metastasis in rectal cancer, such as tumor stage, extramural venous invasion, and carcinoembryonic antigen, to further improve model performance and enhance its clinical applicability.

Previous studies on transrectal ultrasound-based radiomics have predominantly focused on prostate cancer patients (26–29). Only a few transrectal ultrasound radiomics studies have targeted rectal cancer. Abuliezi et al. (30) developed a radiomics model based on CETRUS to predict the efficacy of neoadjuvant therapy in rectal cancer patients, demonstrating certain predictive capability. In this study, we utilized imaging of primary rectal tumors obtained via CETRUS to construct a deep learning radiomics model for predicting distant metastasis in rectal cancer patients. This model showed promising predictive performance and, to some extent, supports the significance of the “seed” in the “seed and soil” hypothesis regarding tumor distant metastasis (31).

In this study, the DenseNet201 architecture was adopted for feature extraction instead of conventional handcrafted radiomics features. Compared with traditional approaches, this data-driven and automated method enables autonomous feature discovery, allowing the identification of latent patterns that are difficult to capture by human observers or predefined mathematical descriptors. Moreover, deep learning models provide hierarchical feature representations, simultaneously capturing low-level texture characteristics and high-level semantic patterns within medical images (32, 33). In addition, by leveraging models pretrained on large-scale datasets such as ImageNet, transfer learning facilitates robust initial feature representations while substantially reducing the burden of extensive feature engineering and parameter tuning commonly required in traditional radiomics workflows, thereby markedly improving analytical efficiency (34, 35). Nevertheless, it should be noted that the inherent “black-box” nature of deep learning features limits their biological interpretability. This trade-off between predictive performance and mechanistic interpretability is a well-recognized challenge in the application of artificial intelligence in medicine.

Among the deep learning radiomics models based on TDUS, CDUS, and CEUS, the CEUS model demonstrated the best performance. This finding aligns with previous radiomics studies based on enhanced CT imaging, where models incorporating contrast agents outperformed those based on non-contrast CT scans (36, 37). A plausible explanation is that contrast agents accentuate intratumoral heterogeneity and tumor microvascular architecture, enabling tumors to exhibit richer information and more complex imaging characteristics. Consequently, during feature selection, the CEUS model retained 101 features, whereas the TDUS and CDUS models retained only 76 and 42 features, respectively. Nevertheless, the precise biological and imaging mechanisms underlying these observations warrant further investigation.

Among the constructed deep learning radiomics models, the TDUS model achieved AUC values of 0.935 and 0.586 in the training and test cohorts, respectively, indicating potential overfitting. This indicates that the model may have predominantly learned dataset-specific noise from the training data, while its ability to capture generalizable features associated with tumor biological behavior remained limited. In future studies, overfitting may be mitigated through strategies such as enhanced regularization, data augmentation, optimization of model architectures, cross-validation, and feature dimensionality reduction.

Compared to the CEUS deep learning radiomics model alone, the combined model that combined clinical factors demonstrated further improvement in AUC values. This suggests that integrating multimodal data can enhance model performance, a conclusion that has also been supported by other radiomics studies (38, 39).

Previous radiomics and deep learning studies aimed at predicting distant metastasis in rectal cancer have primarily focused on CT- or MRI-based imaging modalities (15, 40–42). To our knowledge, this study is the first to explore the potential of CETRUS for predicting distant metastasis in rectal cancer patients. The results demonstrated that the predictive performance of the CETRUS-based model was comparable to that of models developed using CT or MRI. Importantly, CETRUS offers several distinct advantages: ultrasound equipment is more widely available than MRI, particularly in resource-limited settings, and is associated with lower examination costs. Furthermore, unlike CT, ultrasound does not involve ionizing radiation, thereby avoiding radiation exposure. Collectively, these advantages suggest that CETRUS-based predictive models hold considerable promise for clinical application in assessing distant metastasis in rectal cancer patients.

In summary, this study preliminarily explored the application value of a deep learning radiomics model based on CETRUS for predicting distant metastasis in rectal cancer patients. The resulting integrated model demonstrated favorable predictive performance for distant metastasis in this patient population. However, there are still some limitations in this study. First, this was a retrospective study, which may inevitably introduce selection bias. Second, the study was conducted at a single center without external test, and thus the model’s generalizability to other institutions remains to be determined. Finally, this study has an important methodological limitation. The diagnosis of distant metastasis was based on imaging findings from contrast-enhanced CT of the chest, abdomen, and pelvis in combination with contrast-enhanced liver ultrasound, rather than histopathological confirmation, which remains the diagnostic gold standard. In addition, the follow-up period was relatively short. These limitations may have introduced two types of bias. First, small or occult metastatic lesions with atypical imaging features may have been missed, resulting in false-negative classifications. Second, a small proportion of benign lesions may have been misclassified as metastatic disease, leading to false-positive conclusions. Consequently, the metastatic status defined in this study may not fully reflect the true metastatic burden of the patients. Future studies should adopt a prospective design with larger sample sizes and longer follow-up periods, and whenever feasible, suspicious metastatic lesions identified on imaging should undergo biopsy or surgical pathological confirmation. Such strategies would help reduce diagnostic misclassification and further enhance the clinical translational value of the proposed models.

## 5 Conclusion

The clinical-deep learning radiomics model based on CETRUS showed promising predictive performance in assessing distant metastasis in rectal cancer patients. This approach has the potential to assist clinicians in developing personalized patient management strategies, pending further validation to confirm its clinical applicability.

## Data Availability

The raw data supporting the conclusions of this article will be made available by the authors, without undue reservation.

## Supplementary material

Supplementary Figure 1 Visualization of the selected deep learning radiomics features derived from different ultrasound modalities. **(A)** TDUS-based features. **(B)** CDUS-based features. **(C)** CEUS-based features.
