# Supplementary figures and images for: Deep learning radiomics models based on contrast-enhanced transrectal ultrasound for predicting distant metastasis in rectal cancer
Source: Front Oncol. 2026 Feb 9;16:1671887. doi: 10.3389/fonc.2026.1671887 (PMC12926103; doi:10.3389/fonc.2026.1671887)

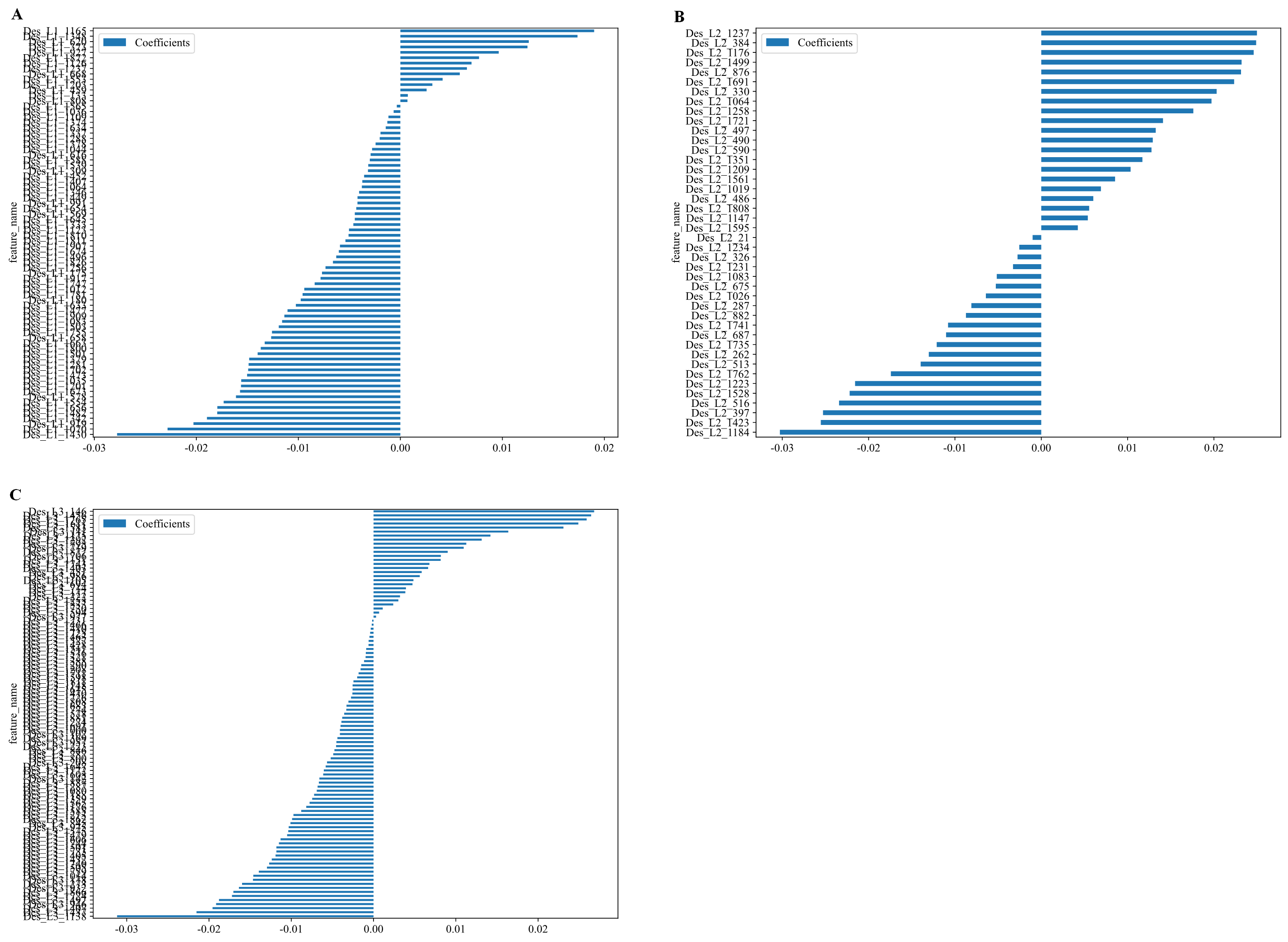

Supplement: Supplementary Figure 1 — Visualization of the selected deep learning radiomics features derived from different ultrasound modalities. (A) TDUS-based features. (B) CDUS-based features. (C) CEUS-based features. [file Image1.tif]
